# Evaluation of Golf Apparel for Ultraviolet Protection: An Analysis of Skin Coverage Provided by Leading U.S. Golf Apparel Brands for a High-Risk Population

**DOI:** 10.7759/cureus.98314

**Published:** 2025-12-02

**Authors:** Taylor Merkle, Jesse Lama, Hayeon Yang, Meera Patel, Erum Ilyas

**Affiliations:** 1 Dermatology, Drexel University College of Medicine, Philadelphia, USA; 2 Biology, University of California, Los Angeles, Los Angeles, USA; 3 Biology, Temple University, Philadelphia, USA

**Keywords:** golf, golf apparel, skin cancer prevention, sun protection, uv exposure, uv-protective clothing

## Abstract

Background

This study aimed to assess the extent of protective design elements and fabric characteristics of apparel items specifically marketed to golfers from leading online U.S. brands for providing adequate ultraviolet (UV) protection across demographic groups, with particular attention to anatomical areas at highest risk of UV exposure.

Methodology

Five leading golf apparel brands were identified using a composite metric. Brand webpages designated for golf-specific apparel were assessed for design features and textile claims across categories (tops, bottoms, accessories, hats) for men, women, and children. A seven-point composite score quantified skin coverage provided by tops. Chi-square tests determined differences in offerings across demographics.

Results

Across 671 golf apparel items, adequate protection of vulnerable body sites was low overall. Ultraviolet protection factor (UPF) labeling was low for clothing options, ranging from 7.1% to 16.7%. Coverage offered by garment design, including the arms, hands, and neck, was limited overall, while chest and back coverage were nearly universal. The mean composite scores for coverage features of tops on a seven-point scale were 3.69 for both men and women and 3.48 for children. Significant differences in top features across demographic groups were observed using a chi-square analyses for back coverage (χ²(2) = 16.59, p < 0.001), shoulder coverage (χ²(2) = 12.40, p = 0.002), whole arm coverage (χ²(2) = 19.85, p < 0.001), and hand coverage (χ²(2) = 7.76, p = 0.021), suggesting design variations among age and gender that may impact sun-protective elements.

Conclusions

Performance apparel specifically marketed toward golfers often lacks key design features and textiles for adequate protection of vulnerable skin. Manufacturers likely emphasize style and wearability over evidence-based UV protective principles; collaboration with dermatologists and public health experts can improve these gaps in garment design and marketing. Clinicians should additionally recognize these shortcomings and counsel golfers on their increased risks, as available clothing options may not ensure adequate UV protection. Recommendation for UPF-certified garments and targeted sun-protective accessories, such as sleeves, can offer more reliable and versatile UV protection for this vulnerable population.

## Introduction

Golfers were 2.4 times more likely to report a history of skin cancer compared to the general population, according to an Australian health survey [1]. With peak play occurring midday during intense ultraviolet (UV) exposure, only 36% of golfers reported “always” wearing sunscreen in a survey of sun-protection strategies [2]. UV radiation exposure to the body during play is estimated to be highest at the shoulders, back, posterior neck, and posterior arms, with comparatively less exposure to the front of the body [3]. These patterns of exposure highlight the role of clothing, through textile and design, as an important means of passive sun protection for the body. Patient education websites broadly recommend selecting dark-hued lightweight fabrics with long-sleeve shirts and pants for optimal UV protection, but fail to offer in-depth options to athletes. As golf courses typically enforce dress code requirements, golfers are subject to limitations in design selection and may rely on choices that are labeled or marketed specifically for golf performance. This study aimed to assess the extent of UV-protective design elements and fabric characteristics in golf-specific marketed apparel across demographic groups, with particular attention to coverage of high-risk anatomical sites.

## Materials and methods

The top five apparel brands specifically marketed to golfers were identified using a composite metric that included Professional Golfers’ Association recommendations, market data through public annual reports, Google search trends, and consumer insights based on aggregated customer reviews and manufacturer claims for ultraviolet protection factor (UPF) ratings. Brands that appeared most frequently across these data sources were designated as the leading golf apparel brands. Exclusion criteria included brands that did not have a specific apparel designation page for golf clothing.

A structured data collection process was conducted by five independent reviewers analyzing each brand’s designated golf apparel webpage over a two-week period between July 7, 2025, and July 21, 2025. For each brand, the number of product offerings was recorded in the following categories: hats, tops, bottoms, and accessories, stratified by gender (men, women) and age group (kids/juniors when applicable). To validate data collection, a second reviewer cross-checked all categories for interrater reliability. Reviewer agreement was high across variables, with discrepancies resolved through consensus with a faculty advisor.

Product design features were further categorized within each category as follows: hats were assessed for wide-brim styles; tops for scalp (hood option), neck (full or partial), back, chest, shoulder, whole arm, and hand coverage; bottoms for whole leg coverage; and accessories for neck, arm, and hand coverage. Partial neck coverage was defined for tops with a fold-down collar, while full neck coverage refers to static mock neck or high-neck designs. Back coverage was defined as coverage of the entire back, excluding the neck. Chest coverage was defined by shirts offering a crew neck, buttons, or a zip to extend coverage of the garment to the base of the anterior neck. Shoulder coverage referenced shirts with a sleeve extending over the entire shoulder, most often short-sleeved shirts, while whole arm coverage was designated shirts with a sleeve extending to the wrist. Hand coverage was designated for shirts offering a thumbhole sleeve or cuff to pull the sleeve over the hand. Accessories were defined as products designated for apparel use outside of hats and clothing during golf, such as removable sleeves or neck gaiters. All products were reviewed for UPF claims. The total number of offerings per feature and category was recorded.

Probabilities were calculated for each feature individually by dividing the number of garments with the feature by the total number of garments. A seven-point composite score for the highest risk exposure areas was assigned to each top by using a point each for incorporating UPF labelling and coverage for the full neck, back, chest, shoulder, whole arm, and hand. The option for a hood was not included in the metric, given dress code restrictions for hooded garments at many golf courses. Mean composite scores were calculated for men’s, women’s, and children’s golf tops to quantify overall protective design. A series of chi-square tests of independence using SPSS (IBM Corp., Armonk, NY, USA) were conducted to examine whether UPF claims and design elements varied across demographics for tops (men, women, and children), with a p-value <0.05 considered statistically significant.

## Results

A total of 671 golf apparel, accessories, and hat items were evaluated across gender and age categories. The presence of UPF labeling was low among clothing groups, ranging from 7.1% in children’s bottoms to 16.7% in children’s tops, while accessories represented 50% and 100% for men’s and women’s, respectively, with limited options being available (N = 4 and N = 2, respectively) (Table 1).

**Table 1:** Total number of apparel options from the golf-specific webpages of top five brands evaluating specific coverage features of tops, bottoms, and accessories with calculated total percentages for men, women, children, and hat options. N represents the total tallied number of items per category, with % within each category calculated by dividing the subtotal by the total number within each category. UPF = ultraviolet protection factor

Feature	Men’s golf tops (N)	Women’s golf tops (N)	Children’s golf tops (N)	Men’s golf bottoms (N)	Women’s golf bottoms (N)	Children’s golf bottoms (N)	Men’s accessories (N)	Women’s accessories (N)	Hats (N)
Total options	228	155	42	76	66	28	4	2	58
UPF rating listed (%)	26 (11.4%)	23 (14.8%)	7 (16.7%)	7 (9.2%)	6 (9.1%)	2 (7.1%)	2 (50%)	2 (100%)	9 (19%)
Full neck coverage (%)	33 (14.5%)	43 (27.7%)	2 (4.8%)	-	-	-	0 (0%)	0 (0%)	-
Partial neck coverage (%)	160 (70.2%)	93 (60%)	34 (81%)	-	-	-	-	-	-
Back coverage (%)	228 (100%)	154 (99.4%)	42 (100%)	-	-	-	-	-	-
Chest coverage (%)	228 (100%)	148 (95.5%)	42 (100%)	-	-	-	-	-	-
Shoulder coverage (%)	215 (94.3%)	123 (79.4%)	37 (88.1%)	-	-	-	-	-	-
Whole arm coverage (%)	112 (49.1%)	73 (47.1%)	15 (35.7%)	-	-	-	4 (100%)	2 (100%)	-
Hand coverage (%)	3 (1.3%)	10 (6.5%)	1 (2.4%)	-	-	-	0 (0%)	0 (0%)	-
Hood option (%)	33 (14%)	16 (10.3%)	4 (9.5%)	-	-	-	-	-	-
Full leg coverage (%)	-	-	-	37 (48.7%)	23 (34.8%)	10 (35.7%)	-	-	-
Wide-brimmed style (%)	-	-	-	-	-	-	-	-	11 (19%)

Among hats, 19% specifically marketed to golfers offered a wide-brim design. Among tops, back and chest coverage was nearly universal, while coverage for the hand ranged from 1.3% to 6.5%, hood options from 9.5% to 14%, and full neck coverage was least common at 4.8% to 27.7%. Shoulder and whole arm coverage were found more frequently among men’s tops, at 94.3% and 49.1%, respectively, compared to women’s, at 79.4% and 47.1%, and children’s, at 88.1% and 35.7%, respectively (Table 1).

A total of six accessories were found with sleeve products, adding coverage for the full arm 100% of the time and for the hands 0% for both men and women. No neck gaiters were identified on the golf apparel pages. Based on the dataset, back coverage was lowest for children, shoulder and whole arm coverage was least for women, and hand coverage was lowest for men (Table 1).

The mean composite score per top on a seven-point scale was 3.69 for men’s, 3.69 for women’s, and 3.48 for children’s. Chi-square tests of design features across demographics for women, men, and children demonstrated significant differences for back coverage (χ²(2) = 16.59, p < 0.001), shoulder coverage (χ²(2) = 12.40, p = 0.002), whole arm coverage (χ²(2) = 19.85, p < 0.001), and hand coverage (χ²(2) = 7.76, p = 0.021) for available options in these groups. No significant differences were found in the presence of UPF claims, full neck coverage, and chest coverage (Table 2).

**Table 2:** Chi-square analysis of independence for the determination of statistically significant differences between men’s, women’s, and children’s apparel tops for providing important design coverage elements and UPF labels from top five golf brands websites. P-value <0.05 was considered statistically significant. UPF = ultraviolet protection factor

	UPF	Full Neck	Back	Chest	Shoulder	Whole Arm	Hand
Chi square test	1.45	1.45	16.59	1.75	12.4	19.85	7.76
p value	0.485	0.485	< 0.001	0.418	0.002	< 0.001	0.021

## Discussion

Data from the 2020 Tokyo Summer Olympic Games identified golf as the second-highest UV radiation exposure risk group among outdoor competitions [4]. With an estimated 47.2 million Americans aged six or older having played the sport in 2024, according to the National Golf Foundation, golfers represent a large and vulnerable population for UV-related skin damage [5]. This exposure aligns with prior data showing a 2.4-fold higher likelihood of reporting skin cancer among golfers [1].

Assessing the most vulnerable body areas exposed to UV radiation can help identify important coverage features pertaining to clothing design. In a study using dosimeters in golfers to assess the standard erythema dose (SED), or the amount of UV exposure that causes reddening of the skin in terms of an estimated annual exposure, the vertex of the head was found to have the most significant SED values [6]. While hats can provide protection, they may not always be worn, according to a prior assessment of sun-protective behaviors among golfers, where participants reported to “always” wearing a hat only 45.5% of the time as a top strategy [2]. Additionally, the effectiveness of hats based on design to protect the skin from UV exposure should be considered, as these can vary based on sun position and environmental conditions. Wide-brimmed hats are generally considered to offer superior coverage to typical baseball-style caps [7]. Results of this study indicate 19% of hats marketed to golfers offered a wide brim style (Table 1). This may represent a missed opportunity to provide added coverage for the neck and ears based on the time of play and angle of sun exposure. Data from a prior study determined that a wide-brimmed hat worn in the summer can reduce the SED to the neck by 25% compared to a baseball-style hat and by almost 50% for the ears [7].

Aside from the vertex of the head, additional vulnerable body sites with significant SED values include the upper back, neck, and forearms, representing further opportunities for protection based on clothing design [6]. When evaluating UVB radiation by polysulphone film dosimeters, a study found that the back of the neck experienced the greatest amount of exposure both under and over clothing. Although collared shirts are a common dress code requirement at many courses, they were shown not to prevent UV exposure to the neck, particularly due to the golfer’s stance, especially when bending at the waist while swinging. The front of the neck was the second-highest exposure under clothing, as most golfers chose to unbutton their collars [3]. In this study, full neck coverage was found in 14.5% of men’s shirts, 27.7% of women’s, and 4.8% of children’s, while partial neck coverage, defined by collared shirts, was found more often in 70.2% of men’s shirts, 60.0% of women’s, and 81.0% of children’s. While the majority of tops offered back and chest coverage at >95.5%, shoulder coverage for women dropped to 79.4% and for children to 88.1% (Table 1). Consideration of mock neck and sleeves in design can provide further protection to these locations.

In addition to body site coverage, it is also important to consider fabric selection, with tightly woven fabrics and darker hues considered more effective at UV protection for the skin [8]. A stated UPF factor offers consumers added validation of the UV-protective properties of the textiles chosen. Despite the potential for textiles to offer reliable UV protection, a survey study found that 70% of respondents stated they would not adopt long sleeve or UV-specialized fabrics due to a perceived lower level of advantages for the style [9]. In this study, clothing specifically advertising UPF was available more frequently to women at 14.8% and children at 16.7% compared to men at 11.4% (Table 1). Given that textiles cannot ensure reliable UV protection without verified UPF testing, the risk of UV exposure to covered skin areas remains significant despite design considerations.

A scoring framework to track textile and design elements was used to evaluate tops for sun-protective features (Table 2). This may help inform future design standards to prioritize skin health in golf apparel. Overall, evaluating the mean composite scores, reflecting textile and design elements for UV protection, showed comparable values for men and women (3.69 on a seven-point scale) and slightly lower for children (3.48). The statistically significant difference across demographic groups for particular design features (back, shoulder, whole arm, and hand) suggests that sun protection may not be the primary concern driving golf top design. Manufacturers may underemphasize explicit UV-protective features because product design is strongly shaped by aesthetic trends, comfort and breathability, production costs, and perceived consumer demand, which can prioritize style and wearability over optimized photoprotection. However, the lack of a statistically significant difference in the presence of UPF claims across demographic groups suggests that this may not be a primary factor influencing demographic-specific consumer preference.

Although overall scores were low, it is important to consider both thermal comfort and UV exposure when designing for this population. This is highlighted by noting that full leg coverage was found in less than half of men’s bottoms at 48.7% and slightly more than a third of offerings for women at 34.8% and children at 35.7% (Table 1). Although golf accessories tended to focus coverage on arms, additional product offerings that are removable or adaptable could provide flexible UV protection without altering garment design or compromising comfort or dress code compliance.

Study limitations included an evaluation of a limited number of clothing brands restricted to online offerings. Different climates and seasons may result in variations of garment availability and modifications of offerings locally available for in-store purchase. Real-world usage was not assessed, potentially resulting in variations in the true photo-protective value of garment selection by golfers. Those at high risk for skin cancer may opt for garments offering more coverage. Other considerations include the selection of clothing from brands not specifically listed on designated golf apparel pages.

## Conclusions

Apparel marketed specifically to golfers often lacks key design features to protect high-risk, frequently exposed areas of the skin. Manufacturers likely prioritize style and wearability in response to consumer demand over adequate photoprotection. This coverage gap represents a missed opportunity to protect a vulnerable population through clothing design, UV-protective textiles, and adaptable or removable accessories when sunscreen use is not optimal. To translate these findings into meaningful improvements in real-world photoprotection, closer collaboration between dermatologists, public health experts, and apparel designers is needed to integrate evidence-based UV-protective principles into garment design and marketing. Clinicians should recognize that relying on golf apparel marketing alone is not sufficient to ensure adequate sun protection when making recommendations to patients. Suggesting targeted sun-protective accessories, such as sleeves, may offer versatility and more reliable UV protection than apparel alone.

## References

[1] Stenner B, Boyle T, Archibald D, Arden N, Hawkes R, Filbay S (2023). Golf participants in Australia have a higher lifetime prevalence of skin cancer compared with the general population. BMJ Open Sport Exerc Med.

[2] Weikert AE, Pagoto SL, Handley E, Courtney JB, Brunke-Reese D, Conroy DE (2021). Golfers' interest in multilevel sun-protection strategies. Int J Environ Res Public Health.

[3] Sung H, Slocum AC (2006). UV radiation exposure to body sites of golfers and effects of clothing. Fam Consum Sci Res J.

[4] Downs NJ, Axelsen T, Schouten P, Igoe DP, Parisi AV, Vanos J (2020). Biologically effective solar ultraviolet exposures and the potential skin cancer risk for individual gold medalists of the 2020 Tokyo Summer Olympic Games. Temperature (Austin).

[5] (2025). Golf industry facts. https://www.ngf.org/the-clubhouse/golf-industry-research/.

[6] Downs NJ, Schouten PW, Parisi AV, Turner J (2009). Measurements of the upper body ultraviolet exposure to golfers: non-melanoma skin cancer risk, and the potential benefits of exposure to sunlight. Photodermatol Photoimmunol Photomed.

[7] Backes C, Religi A, Moccozet L, Vuilleumier L, Vernez D, Bulliard JL (2018). Facial exposure to ultraviolet radiation: predicted sun protection effectiveness of various hat styles. Photodermatol Photoimmunol Photomed.

[8] Lu JT, Ilyas E (2022). An overview of ultraviolet-protective clothing. Cureus.

[9] Sung H, Slocum AC (2004). Golfers’ intention to adopt UV specialized clothing as innovation: based on Rogers theory. J Korean Soc Cloth Text.

